# Longitudinal characterization of dysfunctional T cell-activation during human acute Ebola infection

**DOI:** 10.1038/cddis.2016.55

**Published:** 2016-03-31

**Authors:** C Agrati, C Castilletti, R Casetti, A Sacchi, L Falasca, F Turchi, N Tumino, V Bordoni, E Cimini, D Viola, E Lalle, L Bordi, S Lanini, F Martini, E Nicastri, N Petrosillo, V Puro, M Piacentini, A Di Caro, G P Kobinger, A Zumla, G Ippolito, M R Capobianchi

**Affiliations:** 1 Virology Laboratory, INMI-IRCCS “L.Spallanzani”, Rome, Italy; 2 Cellular Immunology Laboratory, INMI-IRCCS “L.Spallanzani”, Rome, Italy; 3 Microscopy Laboratory, INMI “L.Spallanzani”, Rome, Italy; 4 Epidemiology Division, INMI-IRCCS “L.Spallanzani”, Rome, Italy; 5 Clinical Division, INMI-IRCCS “L.Spallanzani”, Rome, Italy; 6 National Laboratory for Zoonotic Diseases and Special Pathogens, Public Health Agency of Canada, Winnipeg, Canada; 7 Division of Infection and Immunity, University College London, London, UK

## Abstract

Data on immune responses during human Ebola virus disease (EVD) are scanty, due to limitations imposed by biosafety requirements and logistics. A sustained activation of T-cells was recently described but functional studies during the acute phase of human EVD are still missing. Aim of this work was to evaluate the kinetics and functionality of T-cell subsets, as well as the expression of activation, autophagy, apoptosis and exhaustion markers during the acute phase of EVD until recovery. Two EVD patients admitted to the Italian National Institute for Infectious Diseases, Lazzaro Spallanzani, were sampled sequentially from soon after symptom onset until recovery and analyzed by flow cytometry and ELISpot assay. An early and sustained decrease of CD4 T-cells was seen in both patients, with an inversion of the CD4/CD8 ratio that was reverted during the recovery period. In parallel with the CD4 T-cell depletion, a massive T-cell activation occurred and was associated with autophagic/apoptotic phenotype, enhanced expression of the exhaustion marker PD-1 and impaired IFN-gamma production. The immunological impairment was accompanied by EBV reactivation. The association of an early and sustained dysfunctional T-cell activation in parallel to an overall CD4 T-cell decline may represent a previously unknown critical point of Ebola virus (EBOV)-induced immune subversion. The recent observation of late occurrence of EBOV-associated neurological disease highlights the importance to monitor the immuno-competence recovery at discharge as a tool to evaluate the risk of late sequelae associated with resumption of EBOV replication. Further studies are required to define the molecular mechanisms of EVD-driven activation/exhaustion and depletion of T-cells.

Ebola virus (EBOV) is one of the most deadly human pathogens, causing a severe hemorrhagic fever syndrome in both humans and non-human primates with fatality rates ranging from 50 to 70%.^1^ The recent outbreak of Ebola Virus Diseases (EVD) in West Africa highlights the pathogenic nature of this virus, the high mortality rates and pandemic potential. To date, there have been over 27 700 cases and >11 280 deaths.^1, 2^ Although EVD is usually an acute illness, increasing evidences exist of persistent infections and post infection syndromes,^3, 4, 5, 6^ highlighting the need to identify immune correlates of a protective immune response.

Defining human immune responses to EBOV infection, pathogenesis and correlates of protection are important for designing effective therapeutic and vaccination interventions. A decrease in lymphocytes has been observed in studies in mice,^7^ non-human primates^8^ and humans,^9^ and is attributed to apoptotic mechanisms.^7, 10^ Persistent B and T-cell activation has been described in four survivors as long as one month after discharge from the hospital, suggesting recurrent antigenic stimulation.^11^ While aberrant immune responses have been described after EBOV infection (reviewed in^12, 13^), and different patterns of inflammatory mediators have been associated with different clinical outcomes,^9, 10, 11, 14, 15, 16, 17^ data on human immune responses to Ebola virus remain scanty, due to difficulties in obtaining sequential samples through the course of illness and to limitations imposed by biosafety requirements for laboratory analyses.

We conducted a longitudinal study aimed to characterize the kinetics of T-cell phenotypes, activation/differentiation profile, autophagic/apoptotic markers and functionality in two EVD patients from soon after symptom onset through their hospitalization until recovery.

## Results

### EBOV viraemia and T-cell subset frequency

**Figure 1:**
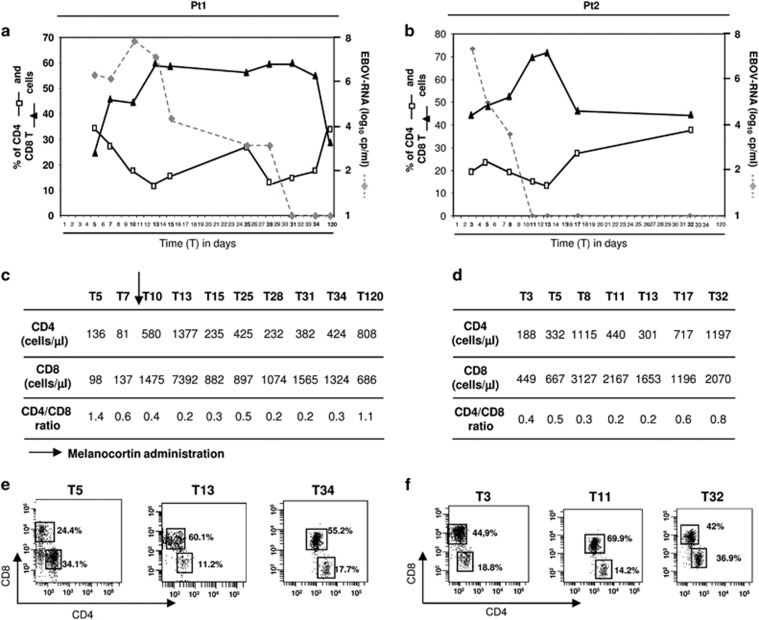
Time course of EBOV viraemia and frequency of CD4 and CD8 T-cells during the course of EBOV infection. The kinetics of viral load (gray dotted line), CD4 (white squares) and CD8 (black triangles) T-cell frequency in pt1 (**a**) and in pt2 (**b**) were analyzed by flow cytometry. The kinetic of CD4 and CD8 T-cell absolute number as well as the CD4/CD8 T cell ratio is shown (**c** and **d**). Time points are presented in days after symptom onset. Representative flow cytometric panels of CD4 and CD8 T-cells in pt1 (**e**) and in pt2 (**f**) are shown. →: melanocortin administration

Figure 1 shows EBOV viraemia over time (gray dotted lines) in patient 1 (pt1, Figure 1a) and in patient 2 (pt2, Figure 1b). Both patients presented with high EBOV viraemia at admission (1.5 × 10^6^ and 2.9 × 10^7^ cp/ml, respectively) but the viral kinetics were markedly different, with a delayed EBOV clearance in pt1 (T31 in pt1 *versus* T11 days from symptom onset in pt2).

As shown in Figures 1a and b, early after infection (T5 for pt1 and T3 for pt2), a low frequency of CD4 T-cells was observed in both patients (pt1: 34.1 and pt2: 18.8%) and persisted during all the course of infection reaching the nadir at T13 (pt1: 11.2 and pt2: 13.1%, respectively); in contrast, CD8 T-cells frequency increased early after infection until T11–13. On the other hand, only during the first days of symptoms this phenomenon resulted in absolute CD4 T-cell count depletion in both patients (136 CD4/μl for pt1 and 188 CD4/μl for pt2); afterwards (at T9 in pt1 and T5 in pt2), a rise in the number of leukocytes was observed that in pt1 was subsequent to melanocortin administration,^18^ while was not treatment-driven in pt2 (Figures 1c and d). Early after infection, a decrease of absolute number of CD8 T-cells was observed only for pt1 (98 CD8/μl) (Figures 1c and d). Moreover, an inversion of the CD4/CD8 T-cell ratio was observed, starting at T7 in pt1 and at T3 in pt2 (Figures 1a and d), with a higher frequency of CD8 (black triangles) in respect to CD4 (white squares) T-cells. Relative to pt2, normalization of the CD4/CD8 T-cell ratio was delayed in pt1 (T120 in pt1 *versus* T32 in pt2). Representative cytometric panels of CD4 and CD8 T-cells subsets at the various time points are shown (Figures 1e and f). Finally, a decrease of B and NK cell frequency was also observed (data not shown).

### Kinetics of T-cell activation

**Figure 2:**
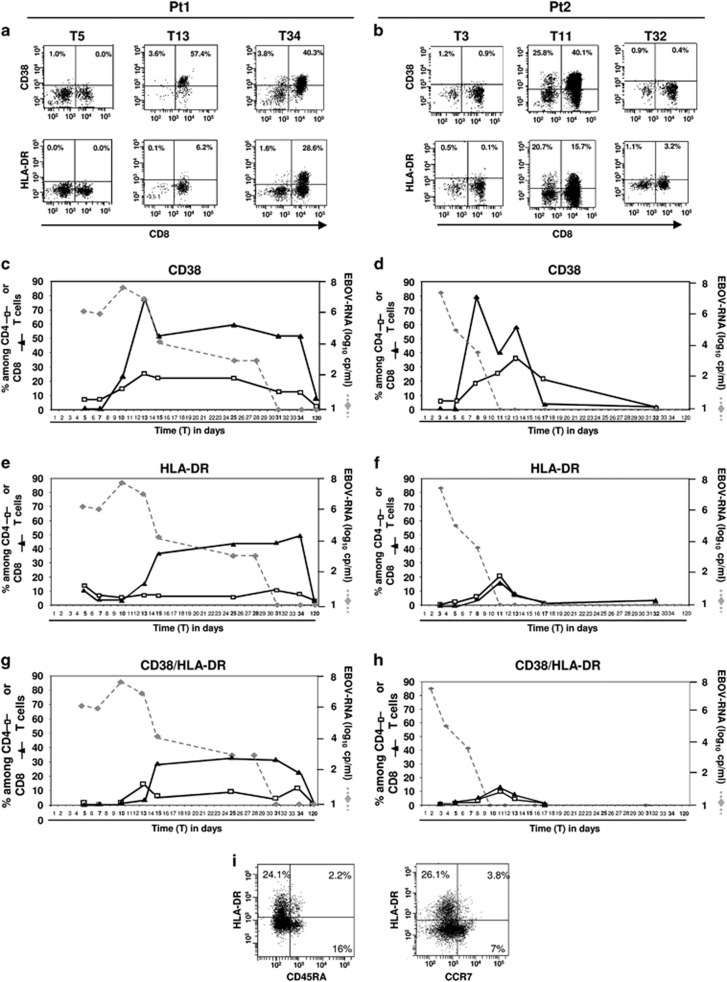
Time course of T-cell activation during EBOV infection. Representative flow cytometric panels showing the kinetics of CD3 T cell activation are shown (**a** and **b**). T-cells were first gated as CD45+, then as CD3+, and finally CD38 or HLA-DR expression in pt1 (**a**) and in pt2 (**b**) is shown at three different time points. The kinetics of CD38 and HLA-DR expression on CD4 and on CD8 T-cells are analyzed by flow cytometry. Data are reported as frequency of CD8 or CD4 T-cells expressing CD38 (**c** and **d**) or HLA-DR (**e** and **f**) or both CD38/HLA-DR (**g** and **h**) among respectively CD8 (black triangle) and CD4 (white square) T-cells. The kinetics of viral load is reported (gray dotted line). Representative flow cytometric panel showing the expression of differentiation markers on activated CD3 T-cells (**i**) are shown

Accordingly to Mc Elroy *et al.*,^11^ the kinetics of T-cell activation was established by monitoring the expression of CD38 and HLA-DR by flow cytometry. Representative dot plots of CD38 and HLA-DR expression on CD3 T-cells at the same time points are shown (Figures 2a and b). The activation of CD8 (black triangles) and CD4 (white squares) T-cells for the entire course of infection is shown in the bottom graphs (Figures 2c and h). In both patients, the frequency of CD38-expressing CD8 T-cells (Figures 2c and d, black triangles) was low in the first days after infection, and then increased, reaching the highest frequency at T13 for pt1 (79.6%) and at T8 for pt2 (79.5%), declining thereafter. A similar trend was also observed for CD4 T-cells (Figures 2c and d, white squares). The analysis of HLA-DR expression on CD8 T-cells revealed a delayed kinetic (Figures 2e and f, black triangles) and a lower activation of CD4 T-cells in pt1 *versus* pt2 (Figures 2e and f, white squares). Moreover, the kinetic of the co-expression of HLA-DR and CD38 presented a profile similar to HLA-DR (Figures 2g and h). Notably, pt1 was characterized by a long-lasting CD8 T-cell activation profile when compared with pt2, possibly due to a more prolonged EBOV viremia (Figures 2c). Indeed, a high frequency of CD8 T-cells expressing both CD38 (51.1%), HLA-DR (49.2%) and CD38HLA-DR (22.3%) was still observed at T34 in pt1 before a decline to just above zero at T120. In contrast, pt2 was characterized by a more rapid decrease of CD8 T-cell activation that became negligible at T17–T32, coinciding with EBOV-RNA clearance from the blood (Figures 2d). Finally, the majority of activated T-cells did not express either CD45RA or CCR7 (Figure 2i), suggesting an effector T-cell phenotype.

### EBOV and T-cell functionality

**Figure 3:**
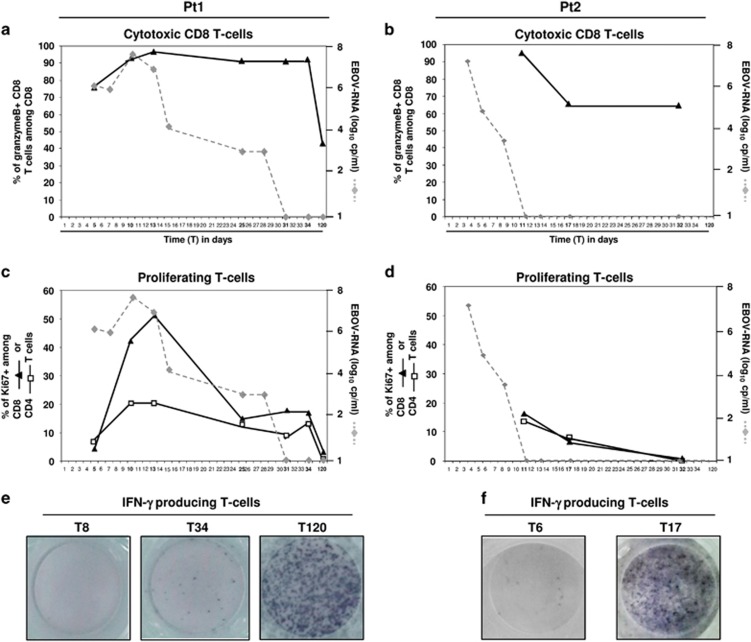
Time course of cytotoxic, proliferating and IFN*γ*-producing T-cells. The kinetics of Granzyme (**a** and **b**) and Ki67 (**c** and **d**) expression on CD8 (black triangles) or CD4 (white squares) T-cells are analyzed by intracellular staining and flow cytometry. The kinetics of viral load is reported (gray dotted line). The Elispot images showing the IFN-*γ* production after PHA stimulation at different time point (T8, T34 and T120 for ot1 and T6, T17 for pt2) are reported (**e** and **f**)

The cytotoxic and proliferating phenotypes of T cells from pt1 and pt2 were analyzed by intracellular expression of the granzyme B (Figures 3a and b) and Ki67 (Figures 3c and d) markers. Data for T3 and T8 time points for pt2 are not available due to an insufficient number of cells recovered to perform all the analyses. The frequency of CD8 T-cells with a cytotoxic profile (Figures 3a and b) was substantially higher in both patients when compared with healthy donors (the average of four healthy donors is shown by hatched line, 46.1%±6). The cytotoxic profile of CD8 T-cells was predominant and also maintained after viral clearance in both patients, dropping down in the convalescent phase (T120 in pt1 and T17 in pt2). Proliferating CD8 and CD4 T-cells were low early after infection, then increased until T11–T13 and dropped down just prior to viral clearance, suggesting an ongoing antigen-driven proliferation (Figures 3c and d). In pt2, the analysis focused only on samples after virus clearance, and a decrease of the proliferating activity of both CD8 and CD4 T-cells was also observed.

Figure 3 show ELISpot images of the IFN-*γ* production after PHA stimulation in pt1 (Figure 3e) and in pt2 (Figure 3f). Poor response, suggestive of functional T-cell anergy, was observed during the viremic phase of the infection from T8 in pt1 and T6 in pt2 with counts at 20 SFC/10^5^ and 95 SFC/10^5^ CD3 cells, respectively, suggesting an impairment of the cell-mediated immune response. The inability of T-cells to produce IFN-*γ* during the acute phase was transient, and reversion occurred, albeit with different kinetics (in pt1 at T34 with 183 SFC/10^5^ and T120 with >2000 SFC/10^5^ CD3 cells; in pt2 at T17 with >2000 SFC/10^5^ CD3 cells), suggesting a temporal association between T-cell anergy and the acute phase of EBOV infection. Notably, the T-cell anergy profile was accompanied by a reactivation of Epstein Barr Virus (EBV) infection, indicated by the presence of EBV-DNAemia both in pt1 (549 000 IU/ml at T13) and in pt2 (10 439 IU/ml at T8).

**Figure 4:**
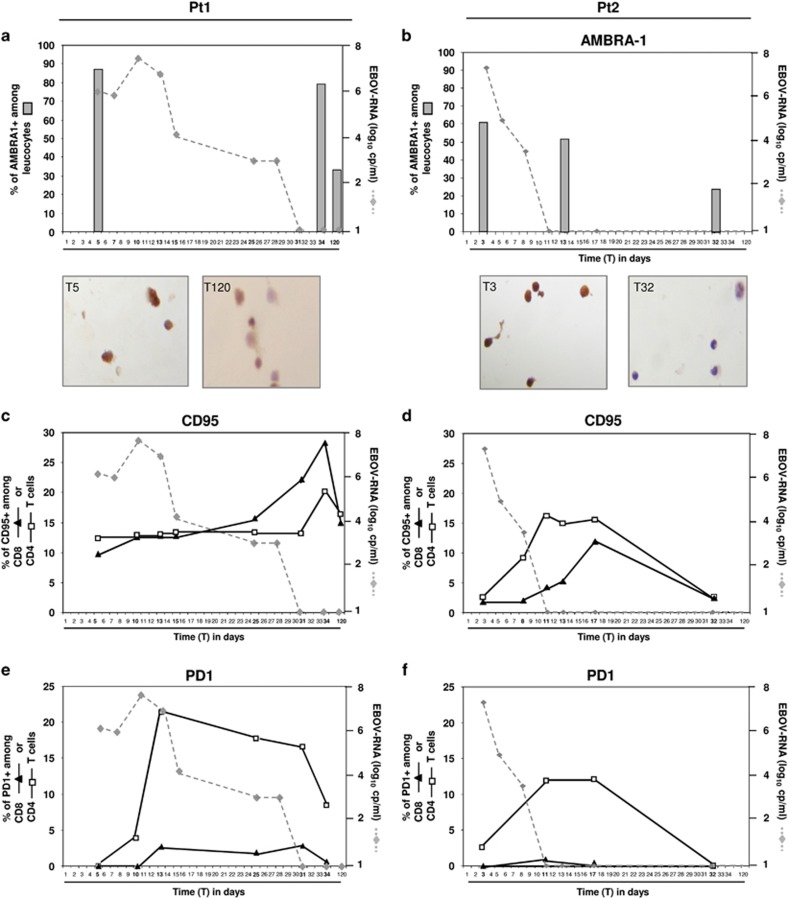
Expression of autophagy, apoptosis and exhaustion markers during EBOV infection. Analysis of AMBRA-1 (**a** and **b**), CD95 (**c** and **d**) and PD-1 (**e** and **f**) expression on leukocytes is shown in pt1 and pt2. AMBRA-1 was analyzed by immunohistochemical assay, while CD95 and PD-1 expression were analyzed on CD8 (black triangles) and CD4 (white squares) T-cells by flow cytometry. Representative images of AMBRA-1 staining are also shown. Data are presented as the percentage of positive cells

In order to identify a potential role for autophagy, apoptosis and exhaustion in the dysfunctional T-cell response during EBOV infection, the expression of AMBRA-1 (Figures 4a and b), CD95 (Figures 4c and d) and PD-1 (Figures 4e and f) on leukocytes was measured in both patients during the viremic phase and after EBOV clearance. Specifically, AMBRA-1 was analyzed by immunohistochemical assay, while CD95 and PD-1 expression were monitored by flow cytometry. Results showed a high percentage of AMBRA-1 positive cells early after infection (T5 in pt1 and T3 in pt2) in both patients (88% in pt1; 62% in pt2), followed by a significant reduction in both patients, although with different kinetics. In fact, AMBRA-1 expressing leukocytes reached low levels at T32 in pt2 (Figure 4b), while in pt1, low values were observed only at T120 (Figure 4a). Representative immuno-histochemical images of AMBRA-1 expressing leukocytes are shown (Figures 4c and d). A transient increase in the level of the CD95 receptor on CD4 (white squares) and CD8 (black triangles) T-cells was detected in both patients (Figures 4c and d), with a higher and delayed kinetic in pt1, suggesting a possible involvement of autophagic/apoptotic pathways in the massive loss of lymphocytes during EBOV infection. Finally, a similar delayed profile was observed when analyzing the PD-1 expression (Figures 4e and f), as a transient increase of PD-1 expression on CD4 T-cell surface was observed in both patients. No significant increase of PD-1 on CD8 T-cells was observed.

## Discussion

Increasing evidences suggest that lymphocytes have an important role to play in the immune response to EBOV.^19, 20^ Studies performed in mice,^7^ non human primates^8^ and in humans during acute EBOV infection,^9^ have reported a loss of lymphocytes. In addition, this loss appears to be more pronounced in fatal cases, suggesting a global suppression of adaptive immunity.^9, 21^ Our study showed a marked reduction of CD4 T-cell frequency to below 20% and a significant increase of CD8 T-cell frequency during the length of the viremic period, which resolved in the convalescent phase. This was accompanied by a persistent inversion of CD4/CD8 T-cell ratio, that was not observed in the mouse model.^7^ The analysis of CD4 and CD8 T-cells absolute numbers in pt1 was affected by the early treatment with melanocortin, inducing a sharp increase of total lymphocytes counts. In contrast, the rise of lymphocyte counts in pt2 in the absence of any treatment is unexplained at present, and may represent an homeostatic reaction to the virus-driven lymphopenia, or, yet, a lymphoprolipherative response to EBV reactivation occurring in both patients. In any case, CD4 T-cells were significantly affected in both patients, while CD8 T-cells decreased only in pt1, whose clinical course was more prolonged and severe.^18^ The marked reduction of the CD4 T-cell population is likely an important driver in the overall loss of lymphocytes. The dramatic loss of CD4 T-cells may limit the initiation and maintenance of effective humoral and cytotoxic T-cell immunity, leading to reversible immunosuppression.

In addition, our findings indicate that EBOV infection induces a sustained CD8 (and to a lesser extent CD4) T-cell activation, which is associated at least in part with an autophagic/apoptotic/exhaustion phenotype and with the impairment of cytokine production, which most likely contributes to the overall decline of functional lymphocytes. The sustained CD8 T-cell activation observed is in accordance with previous data.^11, 21, 22^ Recently, McElroy *et al.*^11^ described a substantial immune activation that persisted also after virus clearance from the blood, suggesting a possible involvement of viral antigen persistence in other compartments.^23^ In our patients, while the frequency of proliferating T-cells diminishes after the virus is cleared from the blood, the expression of activation markers, mainly involving CD8 T cells, is long lasting, at least in pt1. This is consistent with a longer EBOV persistence in the blood and in his seminal compartment where EBOV was detected for up to six months from disease onset (data not shown).

In animal models, there are observations supporting the hypothesis that filovirus-specific CD8 T-cells are contributing to viral control and clearance.^24, 25^ Substantial evidence, particularly data from vaccination studies,^20^ indicate a role for CD8 T-cells in the immediate control of filovirus infection, while B and CD4 T cells participate in the ultimate long-term control (and possibly clearance) of virus replication.^26^ We observed a high frequency of granzyme B-expressing CD8 T-cells during the active replication phase and after clearance of EBOV viremia. The analysis of IFN-*γ* production in response to mitogenic stimulation also revealed an almost complete immunological anergy that was reversed in the convalescent phase. Functional anergy is a commonly observed feature in patients who show aggressive course of several viral infections (i.e. HIV, influenza^27, 28^), and an immunosuppressive capability of EBOV has been proposed.^12, 29, 30, 31, 32^ We performed a multiple analysis focused to identify the molecular pathways possibly involved in T-cell loss and impairment (e.g. autophagy, apoptosis and exhaustion). The ability of EBOV to induce lymphocyte apoptosis was already described *in vitro* and *in vivo*.^7, 8, 9, 33^ In contrast, no data are available on AMBRA-1 and PD-1 involvement in Ebola pathogenesis. Our results showed that all these parameters increased during EBOV infection, with an early induction of AMBRA-1 and a slightly delayed increase of CD95 and PD-1 occurring at the time of EBOV viremia reduction. Of note, PD-1 increase was a specific feature of CD4 T-cells, suggesting a PD-1 driven exhaustion of T helper immune functions. Interestingly, the immunosuppression observed in both patients coincided with the reactivation of EBV replication in the blood, a phenomenon common to other immunosuppressive conditions.^34, 35^ Recently, increasing evidences exist of EVD persistence in several compartments, and emerging cases of late-onset disease were reported,^3, 4, 5, 6^ suggesting the importance to identify immunological correlates of sterilizing immunity.

Two different models for the immune response to Ebola infection have been proposed.^22^ Our data support a combination of both models, as we simultaneously observed a strong immunosuppression (leading to EBV reactivation) as well as CD8 T-cell activation that gradually outpaced the EBOV RNA from the blood. The patients received a number of treatments, including experimental drugs (see methods section). As in most cases described until now outside of Africa,^36^ the treatments received by the patients were not uniform, so it is not possible to dissect their contribution of in these phenomena. In particular, the antiviral activity of Favipiravir was different in the two patients as the viral load in pt1 was still increasing during the treatment while in pt2 was decreasing from treatment initiation. Accordingly, the kinetics of immune alterations was very different in the two patients. In pt2, the decreased viral load was associated with low levels of proliferating T cells and decreased levels of granzyme-expressing CD8 T cells. However, the decline of viral load did not prevent CD4 loss and the increased expression of activation markers. In pt1, the proliferation rate of T cells followed the kinetics of viral load, while the cytotoxic T cells persisted as long as the virus persisted.

Our study shows for the first time that human EVD is associated with an early, deep and sustained immune dysfunction that reverts during the recovery period. The association of an apparent early dysfunctional T-cell activation in parallel to an overall decline of lymphocytes, particularly of the CD4 compartment, may represent a critical point of EBOV-induced immune subversion and highlight the complex virus/host interactions of this pathogen. Further studies to define the molecular mechanisms of EBOV-driven activation/anergy and depletion of T-cells are required.

## Materials and Methods

### Ethical approval

The study was approved by the local Ethics Committee (approval number: 14/2015), and written informed consent was received from participants prior to inclusion in the study.

### Patients and sampling

In November 2014 and May 2015, respectively, two male patients (pt1: 51 years old and pt2: 37 years old) with EVD were admitted at the National Institute for Infectious Diseases (INMI) Lazzaro Spallanzani, in Rome, Italy.^18, 37^ Pt1 was admitted at day 5 (T5) and pt2 at day 3 (T3) after onset of symptoms, respectively. Pt1 was treated from T5 with high dose oral favipiravir for 4 days, two EVD convalescent plasma bags (at T6 and at T10), one i.v. infusion of melanocortin (at T9) and two i.v. infusions of ZMab (at T12 and at T14). Pt2 was treated from T3 with high dose oral favipiravir for 10 day and received two i.v. infusions of Mill77 at T4 and at T7. Peripheral blood samples were collected sequentially in EDTA-blood collection tubes (BD Vacutainer, New Jersey, USA) from admission to recovery, at different time points (over 120 days for pt1 and 32 days for pt2).

### Leukocytes isolation

All assays on specimens taken during the acute phase, harboring potentially infectious virus, were performed in the BSL-4 facility at INMI. Leukocytes were isolated by lysing red cells with NH4Cl (Sigma-Aldrich, St. Louis, MS, USA), then washed in RPMI-1640 medium (Sigma-Aldrich, St. Louis, MS, USA) supplemented with 10% heat inactivated FCS (Euroclone, Italy) and stored at −80 °C in 90% FCS/10% DMSO (Euroclone, Italy). Cryopreserved leukocytes were rapidly thawed, washed with PBS and fixed with 10% paraformaldehyde (PFA) for 30 min at room temperature, to achieve virus inactivation. Fixed samples were transferred to BSL-2 laboratory for subsequent work.

### Antibodies and flow cytometry

Flow cytometry procedures have been standardized in our laboratory allowing staining 10%-PFA fixed samples in BSL-2 laboratory. Fixed leukocytes were washed twice with RPMI-1640 medium and then stained with conjugate anti-human monoclonal antibodies (mAbs) able to determine activation, exhaustion and apoptotic status. Specifically, CD45-HorizonV500 (clone 2D1), CD3-FITC (clone UCHT1), CD3-HorizonV500 (clone UCHT1), CD4-HorizonV450 (RPA-T4), CD8-APC-H7 (clone SK1), CD8-PerCP (clone SK1) and CD38-HorizonV450 (Clone HB7) mAbs were purchased from BD Biosciences; CD3-PerCP-Cy5.5 (clone SP34-2), CCR7-PE-CY7 (clone 3D12), CD95-APC (clone DX2), HLA-DR-PE (clone G46-6), CD45RA-APC (clone HI100), PD-1 PE (clone MIH4), mAbs were purchased from BD Pharmigen (San Jose, CA, USA). Isotype-matched control mAbs were used in all experiments. 5 × 10^5^ PFA-fixed leukocytes were incubated for 15 min at 4 °C with the indicated mAbs. After washing (PBS/1%BSA/0.1% sodium azide), samples were immediately analyzed using a FACSCanto II flow cytometer.

Cytotoxicity and proliferation markers were detected by intracellular staining for Granzyme B (clone GB11) and Ki67 (clone B56) (BD Biosciences, San Jose, CA, USA)). Briefly, fixed leukocytes were stained with mAbs specific for membrane antigens as described above; then the leukocytes were washed and incubated with antibodies against Granzyme B and Ki67 in a permeabilization buffer (PBS/1%BSA/0.5% saponin) for 20 min at room temperature. Finally the cells were washed in wash/permeabilization buffer (PBS/1%BSA/0.1% saponin), and immediately analyzed on a FACSCanto II flow cytometer. A total of 100 000 events were acquired for each sample and analyzed with Diva software (BD Biosciences). Leukocytes from healthy donors were stained as controls. Raw data will be available upon request.

### IFN-γ production by ELISpot assay

IFN-γ production was evaluated in BSL-4 laboratory after mitogenic stimulation. Specifically, leukocytes from patients in viremic and non-viremic phases were thawed, counted by Scepter counter (Millipore) and seeded at 3x10^5^cells/well in RPMI-1640 medium (Sigma-Aldrich, St. Louis, MS, USA) supplemented with 10% pre-tested heat inactivated FCS (Euroclone, Italy). Cells were then stimulated with phytohemagglutinin (PHA, 5 mg/ml) for 20 h, and the immunological competence was evaluated by IFN-γ enzyme-linked immunospot assay (ELISpot assay, AID Diagnostika, Germany). Leukocytes from healthy donors were used as internal positive controls. CD3 T-cells count from the same samples was evaluated by flow cytometry as described above, and the results of the ELISpot assay were then normalized as spot forming cells (SFC)/10^5^ CD3.

### Immunohistochemical analysis

Leukocytes were fixed with 10% PFA, washed and were incubated with 3% H_2_O_2_ for 5 min to block endogenous peroxidase activity. Nonspecific antibody binding was reduced by incubation with normal goat serum. The primary antibody utilized was a rabbit anti-AMBRA1 (ProSci, Poway, CA). Reaction was visualized using a streptavidin-biotin-immunoperoxidase system with DAB (Biogenex, Fremont, CA) as chromogen substrate. Negative control staining was performed. Cells were counterstained in Mayer's acid hemalum. Leukocytes were counted under a light microscope by using a 40x objective with a field diameter of 0.52 mm. For each slide, a minimum of 10 fields were examined. The results are expressed as percentage of positive/total leuckocytes.

## Abbreviations

EVD: Ebola Virus Disease
EBOV: Ebola Virus
EBV: Epstein Barr Virus
PFA: Paraformaldehyde
